# An extract of *Urtica dioica* L. mitigates obesity induced insulin resistance in mice skeletal muscle via protein phosphatase 2A (PP2A)

**DOI:** 10.1038/srep22222

**Published:** 2016-02-26

**Authors:** Diana N. Obanda, David Ribnicky, Yongmei Yu, Jacqueline Stephens, William T. Cefalu

**Affiliations:** 1 Pennington Biomedical Research Center, Louisiana State University, Baton Rouge, LA 70808, USA; 2 Department of Plant Biology, Rutgers University, New Brunswick, NJ 08901, USA.

## Abstract

The leaf extract of *Urtica dioica* L. (UT) has been reported to improve glucose homeostasis *in vivo*, but definitive studies on efficacy and mechanism of action are lacking. We investigated the effects of UT on obesity- induced insulin resistance in skeletal muscle. Male C57BL/6J mice were divided into three groups: low-fat diet (LFD), high-fat diet (HFD) and HFD supplemented with UT. Body weight, body composition, plasma glucose and plasma insulin were monitored. Skeletal muscle (gastrocnemius) was analyzed for insulin sensitivity, ceramide accumulation and the post translational modification and activity of protein phosphatase 2A (PP2A). PP2A is activated by ceramides and dephosphorylates Akt. C2C12 myotubes exposed to excess free fatty acids with or without UT were also evaluated for insulin signaling and modulation of PP2A. The HFD induced insulin resistance, increased fasting plasma glucose, enhanced ceramide accumulation and PP2A activity in skeletal muscle. Supplementation with UT improved plasma glucose homeostasis and enhanced skeletal muscle insulin sensitivity without affecting body weight and body composition. In myotubes, UT attenuated the ability of FFAs to induce insulin resistance and PP2A hyperactivity without affecting ceramide accumulation and PP2A expression. UT decreased PP2A activity through posttranslational modification that was accompanied by a reduction in Akt dephosphorylation.

Obesity induced insulin resistance is a key pathophysiologic feature of type 2 diabetes mellitus (T2DM). Progression of insulin resistance into overt T2DM can be prevented or delayed by timely intervention^1^,^2^. Treatment of subjects with insulin resistance is directed at increasing insulin sensitivity. Reduction in calorie intake and increase in physical activity are effective but difficult to maintain in the long-term. Therefore, conventional medications that address insulin resistance are an option in clinical medicine^3^ and are a major focus for drug development.

Insulin resistance is induced by the accumulation of lipid metabolites from excess fat in insulin sensitive cells. Excess dietary carbohydrate not stored as glycogen or oxidized for energy is converted to fat by *de novo* lipogenesis^4^. Among the fatty acid derived metabolites, ceramides are most active in negatively regulating insulin signaling^5^,^6^. The primary target of ceramides in the insulin signaling pathway is Akt whose activity state is regulated by serine/threonine phosphorylation^5^,^6^,^7^. Ceramides activate cytosolic protein phosphatase 2A (PP2A) a serine/threonine phosphatase that dephosphorylates Akt leaving it inactivated and unable to mediate insulin stimulated glucose uptake and metabolism^7^. Thus, interventions that may modulate this pathway may be useful for treatment for insulin resistance.

Because some conventional medications marketed for insulin resistance have troublesome side effects^8^,^9^, there is increasing interest in natural complementary and alternative therapies from plants. Use of over the counter botanical agents is extensively practiced by a large number of patients. Plants have traditionally been a rich source of medicinal compounds for many indications. A number of botanicals have shown promise as therapies for insulin resistance and hyperglycemia. Among these is the extract of *Urtica dioica* L. (UT) or stinging nettle (family Urticaceae) whose blood glucose lowering effect has been historically noted and supported by recent animal studies and *in vitro* studies^10^,^11^,^12^,^13^. However, the mechanism(s) that contribute to these positive systemic effects of UT are largely unknown and bioactive components are not elucidated. Despite this paucity of data on efficacy and mechanism(s), UT dietary supplements are widely available in the United States. The acceptance and widespread use of botanical supplements by the general public justifies research in investigating their mechanisms of action. The overall objective of the current study was to examine the efficacy and mechanism(s) of action of UT as a dietary supplement for ameliorating diet induced insulin resistance in skeletal muscle.

In this study, we hypothesized that UT maintains insulin sensitivity through mechanisms that enhance Akt phosphorylation despite the accumulation of ceramides in skeletal muscle. Increased Akt phosphorylation can be achieved by either increase in the activities of kinases that phosphorylate it or by a decrease in activities of phosphatases that modulate its activity. Based on previous reports that have demonstrated that PP2A inhibition with okadaic acid or a neutralizing antibody increased Akt activity^14^,^15^,^16^,^17^, we determined the effect of UT on the activity of PP2A. Our observations from two model systems that include *in vitro* and *in vivo* studies demonstrate that supplementation with UT attenuates lipid induced PP2A hyperactivity and enhances insulin sensitivity and insulin stimulated glucose metabolism in skeletal muscle. These studies strongly suggest that UT has metabolically beneficial effects in skeletal muscle and indicate its potential as a botanical supplement for metabolic disease states such as insulin resistance.

## Materials and Methods

### Source and preparation and of plant extract

*Urtica dioica* L. (UT) was harvested as the total herb above the root mass and a voucher specimen was produced by the Botanical Research Center and deposited in the Chrysler Herbarium at Rutgers University. The plant was harvested in New Jersey. Geographic coordinates of the botanical collection site are 40°36′03.9“N 74°40'30.1“W. The freeze-dried plant was stored at −20 °C. For extraction, 3 kg of dried plant was heated with 15 liters of 80% ethanol (v/v) to 80 °C for 2 h and allowed to continue to extract for an additional 10 hours at 20 °C. The extract was filtered through cheesecloth to remove particulates and ethanol was removed by rotary evaporation to less than 1 liter of final extract. The aqueous extract was then freeze dried from −48 to 20 °C in a glass tray and then homogenized using a mortar and pestle.

### Animals

All experiments and procedures were performed in accordance with relevant guidelines and regulations of the Pennington Biomedical Research Center. All animal experiments and procedures were performed in accordance to a protocol approved by the Institutional Animal Care and Use Committee (IACUC) of the Pennington Biomedical Research Center. Thirty three (33) male C57BL/6J mice at 5 weeks age were ordered from Jackson Laboratories, Inc. (Bar Harbor, Maine, USA). After one week quarantine followed by baseline feeding of all mice for an additional week on the low fat diet (Research Diets D12450B), mice were randomly divided into a low-fat diet group (LFD as negative control, n = 11), high-fat diet group (HFD, Research Diets D12492) as positive control, n = 11) and HFD supplemented with UT group (n = 11). UT extract was incorporated into the high-fat diet at 0.5% w/w. Mice were singly housed in shoebox cages with corncob bedding in controlled environmental conditions (22 °C), 12 hour light dark cycle with *ad libitum* access to food and water. Food intake (the difference of weight administered and leftover plus spillage) and body weight were monitored and recorded weekly.

### Body composition measurement

Body composition was determined by nuclear magnetic resonance (NMR-Bruker, Newark, DE, USA) as previously shown^18^. Body fat mass, muscle mass and free fluid were recorded at baseline (week 0) and at weeks 6 and 12 of feeding.

### Blood chemistry

Fasting (4h) plasma glucose and insulin were determined at baseline and after 6 and 12 weeks of treatment. Insulin levels were determined by a mouse insulin enzyme linked immunosorbent assay (ELISA) kit (Crystal Chem, Downers Grove, IL). Blood glucose was measured using a portable glucometer (Milipitas, CA, USA). The intraperitoneal insulin tolerance test (IPITT) and intraperitoneal glucose tolerance test (IPGTT) were performed at week 10 and week 12, of feeding respectively as described previously^18^. For glucose tolerance tests, mice were fasted for 6 h prior to injections of 2 g glucose/kg body weight. For insulin tolerance tests, mice received 0.5 U of insulin/kg body weight (Humulin; Eli Lilly, Indianapolis, IN). Blood glucose concentrations were measured on the nicked tail vein at time 0 (baseline), 15, 30, 60 and 120 min after glucose or insulin injections. HOMA-IR was used as a measure of insulin resistance and was calculated from fasting insulin and glucose levels. Using the trapezoidal method, the area under curves (AUC) showing the change of blood glucose level over the course of IPGT and IPIT were calculated.

### Tissue collection and processing

At 18 weeks of age, following 12 weeks of treatment each group was divided into basal and insulin-stimulated subgroups. The basal sub group (n = 5), were given an intraperitoneal injection of saline after 6 hours fasting while the insulin-stimulated sub group (n = 6) was given insulin at a dose of 2 U/kg body weight. After 10 minutes, animals were euthanized by CO_2_ inhalation followed by cervical dislocation and decapitation. Blood and tissue were snap frozen in liquid nitrogen and stored at −80 °C for later analysis.

### Tissue processing and immunoblotting

50 mg gastrocnemius muscle was dissected and homogenized in Buffer A (1% Triton X-100, 20 mmol Tris (pH 75), 2.5 mmol sodium pyrophosphate, 150 mM NaCl, 1 mmol EGTA, 1 mmol sodium vanadate, 2 mM beta-glycerophosphate, 1 μg/ml leupeptin, 1 ul/ml aprotinin, 1 ul/ml PMSF) using a PRO 200 homogenizer (PRO Scientific, Oxford, CT, USA). Samples were centrifuged (12000 rpm, 10 min at 4 °C) and protein concentrations of the supernatant determined by the Bio-Rad protein assay kit (Bio-Rad laboratories, Inc. Hercules, CA). Supernatants (80 μg) were resolved by SDS-PAGE and subjected to standard immunoblotting. Protein abundance was detected with antibodies against, Akt1, Akt2, phospho-Akt (ser 473), phospho-Akt (Thr 308), AMPKα1, AMPKα2, phospho AMPK (Thr 172) (Millipore, Temecula, CA). Other antibodies were PP2A-A, PP2A-B, PP2A-C (Cat No. SC-80665), methyl PP2A-C (Cat No. SC-81603) and phospho PP2A Tyr 307 (Cat No. SC-12615) all from Santa Cruz Biotechnology, Santa Cruz, CA). Protein expression levels were normalized by tubulin (Santa Cruz Biotechnology, Santa Cruz, CA). Phosphorylation or methylation levels were normalized by the corresponding protein expression. Optical densities of protein bands were analyzed using Image J. Data are expressed as the fold difference of the LFD control group.

### Cell culture and *in vitro* assays

C2C12 myoblasts (ATCC; #CRL-1771) were maintained at 37 °C, 95% air and 5% CO_2_ in high glucose DMEM supplemented with 10% FBS serum and antibiotics. For individual experiments, myoblasts were sub-cultured onto 6 well plates, grown to 100% confluence and differentiated into fused myotubes for 4 days by switching to media with 2% horse serum. All cells used were within 5 passages. For cell culture experiments, the dried extract was solubilized in 100% dimethyl sulfoxide (DMSO) at a concentration that was 1000-fold higher than experimental concentrations and then diluted into the cell media. A final concentration of 1–10 ug/ml was used to treat cells. Cultures were exposed to FFAs (palmitic acid) conjugated to 1% bovine serum albumin (BSA) and constituted to a final concentration 250 μM with or without 5 ug/ml UT extract or 10 nM okadaic acid (Millipore, Temecula, CA, USA) for 16 hours. For western blotting, 100 nM insulin was added 8 minutes before subsequent collection and analysis. Standard western blotting was performed using 80 μg of cell extracts. Specific antibodies used were the same as those shown above. In all cases, western blot results were verified in three separate experiments. Data are expressed as the fold difference of the vehicle control. The glycogen accumulation assay was performed as described previously^10^. Results were normalized by protein concentration measured by Bio-Rad protein assay kit (Bio-Rad Laboratories, Hercules, CA), and glycogen content was presented as glucose equivalent per well. To determine glucose uptake, after washing with PBS, cells were exposed to 0 or 100 nM insulin in Krebs-Ringer HEPES (KRH) buffer for 15 min followed by an additional incubation for 5 min with 2-deoxy glucose (100 μM, 0.5 μCi). Cells were washed four times with ice-cold KRH buffer, lysed in 250 μl of 0.05 N NaOH and then transferred to vials with scintillation cocktail. Radioactivity in the cells was measured by liquid scintillation counter.

### Ceramide Quantification

Lipids were extracted from 30 mg skeletal muscle or cell lysates corresponding to 300 ug protein and total ceramides quantified by tandemn mass spectrometry (LCMS/MS) as previously described^5^.

### PP2A activity

PP2A activity was assayed in lysate corresponding to 300 ug protein processed from cells or muscle tissue. Lysis buffer contained 20 mM imidazole-HCl, 2 mM EDTA, 2 mM EGTA, pH 7.0 with 10 mg/mL each of aprotinin, leupeptin, pepstatin, 1 mM benzamidine, and 1 mM PMSF. Tissue was homogenized on ice and centrifuged at 12,000 rpm for 10 minutes at 4 °C. Cells were sonicated before centrifuging similarly. The supernatants were used to assay PP2A phosphatase activity by a standard kit (EMD Millipore, Temecula CA) according to the manufacturer’s instructions. The intensity of the color reaction was measured at 650 nm on a Bio-rad microplate spectrophotometer.

### Statistical analyses

Data are expressed or graphed as mean ± SEM. Statistical significance was determined by comparing means by analysis of variance (ANOVA). Significant differences observed were followed up using the Fisher’s least significant difference (LSD) test. All tests used P < 0.05 as the level of statistical significance.

## Results

### Supplementation of HFD with *Urtica dioica* L. does not alter food intake, body weight or body composition

At baseline at six weeks of age, body weights were not statistically different in the three groups. At week 12 the weights had increased by 27.1% in the LFD group, 63.6% in the HFD and 58.4% in the HFD + UT group (Table 1). The HFD and HFD + UT were not significantly different (p = ns, Table 1) but, both were significantly different from LFD (LFD vs HFD, P < 0.01; LFD vs HFD + UT, P < 0.01). Daily food consumption was higher in LFD group but was not different between HFD and HFD + UT groups (Table 1). As expected, body composition was similar in the three groups at baseline. After 12 weeks feeding, there was a 12.7% increase in fat mass with LFD, 82.4% increased for the HFD, and 91.7% in the HFD + UT group. Notably, fat mass was not statistically different between HFD and HFD + UT groups. After 12 weeks of treatment, the decrease in percent muscle was 1.49%, for the LFD group, 14.8% for the HFD and a 15.4% decrease was observed in the HFD + UT group. There were no statistical differences in percent muscle between HFD and HFD + UT groups (Table. 1). Free fluid mass was not different between the three groups and did not change over the 12 weeks (data not shown).

### Supplementation of HFD with *Urtica dioica* L. enhances glucose disposal and insulin signaling *in vivo*

**Figure 1:**
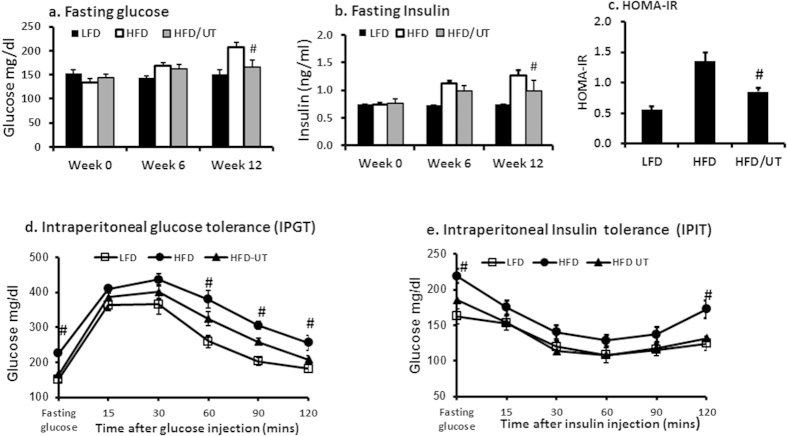
UT extract significantly enhanced plasma glucose homeostasis. Male 6 week old C57BL/6J mice were fed the respective experimental diets for 12 weeks. After a four hour fast at weeks 0, 6 and 12, **(a)** blood glucose was determined using a portable glucometer and **(b)** insulin was determined by an ultrasensitive mouse insulin enzyme linked immunosorbent assay (ELISA) kit. **(c)** HOMA-IR at week 12 was calculated from fasting insulin and glucose levels. **(d)** The Intraperitoneal glucose tolerance test (IPGT) was performed at week 12. Mice were fasted for 4 h prior to injections with 2 g glucose/kg body weight. Blood glucose concentrations were measured on the nicked tail vein at the indicated time frames after glucose injection. **(e)** The intraperitoneal insulin tolerance test (IPIT) was performed at week 10. Mice were fasted for 4 h prior to injections with 0.5 U of insulin/kg body weight. Blood glucose concentrations were measured on the nicked tail vein at the indicated time frames after glucose injection. Data are mean ± SEM (n = 11). ^#^denotes significant difference between HFD and HFD + UT (P < 0.05).

Fasting plasma glucose and insulin were not different between the three groups at baseline but were significantly increased in the HFD group by week 12 (Fig. 1a,b). Insulin resistance (HOMA-IR) was increased in the HFD (Fig. 1c). Supplementation of the HFD with UT significantly lowered fasting glucose and insulin and improved insulin resistance as assessed by HOMA-IR (HFD vs HFD + UT, P < 0.01; Fig. 1a–c). In a glucose tolerance test, blood glucose levels for the HFD + UT group were significantly lower than those of the HFD group at the 60, 90 and 120 minute intervals (P < 0.01;Fig. 1d). However, both the HFD and HFD + UT groups had significantly higher glucose at these time intervals when compared to the LFD group. In an insulin tolerance test, blood glucose levels in the HFD + UT group were significantly lower than those of the HFD only at the 120 minute interval (P < 0.01) and were not different from LFD group at all other time intervals (Fig. 1e). The rate of decline of glucose between baseline and 60 minutes after insulin injection was 2.2, 1.3 and 1.54% per minute for LFD, HFD and HFD/UT respectively and was not statistically different between the HFD and the HFD/UT groups. The area under curves (AUC) for glucose tolerance test was not different between the HFD and the HFD/UT groups. However the AUC for insulin tolerance test curve was significantly lower in the HFD/UT group compared to the HFD group (Table 2).

**Figure 2:**
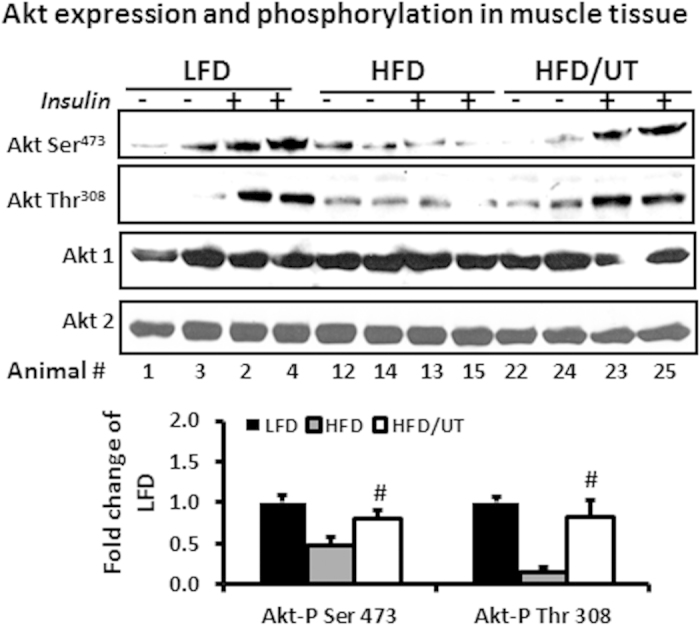
UT extract enhances insulin signaling in skeletal muscle tissue. Male C57BL/6J mice were fed the experimental diets for 12 weeks. Ten minutes prior to sacrifice, mice were injected with 2 U/Kg body weight insulin or saline. Protein homogenates from the gastrocnemius muscle were separated by SDS-PAGE and analyzed by immune blot analysis. Representative blots for Akt phosphorylation and expression are shown. Fold change relative to LFD alone for each Akt phosphorylation was calculated and mean ± SEM graphed (n = 11). ^#^denotes significant difference between HFD and HFD + UT (P < 0.05).

To assess potential direct effects on skeletal muscle, insulin signaling was examined in gastrocnemius muscle from these mice. As shown in Fig. 2, HFD feeding was accompanied by a loss of insulin stimulated Akt serine and threonine phosphorylation. Supplementation of the HFD with UT significantly enhanced insulin stimulated Akt serine and threonine phosphorylation (P < 0.01; Fig. 2). There were no differences in Akt 1 and Akt 2 protein expression levels among the three groups. UT supplementation had no effect on IRS, insulin receptor protein expression, or P13K and AMPK protein expression and activity (data not shown).

### Supplementation of *Urtica dioica* L. to FFA treated C2C12 myotubes reduces the effects of FFA on insulin signaling, glucose uptake and glycogen synthesis

**Figure 3:**
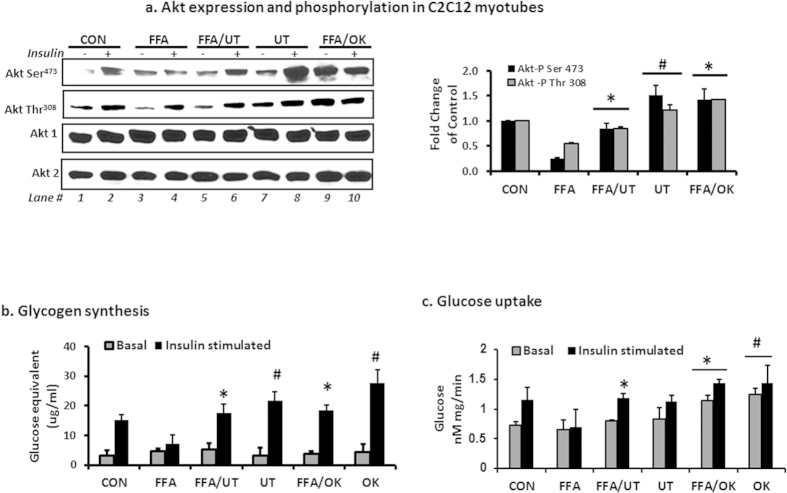
UT extract enhances insulin signaling and glucose uptake in skeletal muscle cells. Fully differentiated C2C12 myotubes were incubated with palmitate (250 uM) with or without UT (5 ug/ml) or 10 nM okadaic acid (OK) for 16 hours and **(a)** myotubes were stimulated with vehicle or 100 nM insulin for 8 minutes. Cell lysates were separated by SDS-PAGE and analyzed by immune blot analysis for Akt serine and threonine phosphorylation and Akt protein expression. The bar chart represents quantification of band density by image J software. **(b)** A second set of cells was used to determine glycogen accumulation with use of glycogen hydrolysis followed by glucose determination in both basal and insulin-stimulated states. Results represent an experiment independently repeated three times on different batches of myotubes. **(c)** A third set of cells was used to determine glucose uptake in both basal and insulin stimulated states by determining radioactivity after uptake of 2-deoxy glucose. *denotes significant difference with FFA only treatment (P < 0.05). ^**#**^denotes significant difference with the control treatment (P < 0.05).

As expected, exposure of myotubes to FFAs induced a decrease in Akt phosphorylation and also decreased insulin induced glycogen synthesis. Co-incubation with FFAs and 5 ug/ml UT or okadaic acid attenuated the ability of FFAs to induce Akt dephosphorylation and reduce glycogen synthesis (Fig. 3a lane 4 vs lane 6 and 10 and Fig. 3b). Incubation with UT only or okadaic acid only enhanced Akt phosphorylation and increased glycogen accumulation compared to the vehicle only treatment (P < 0.05; Fig. 3a lane 2 vs lane 8 and Fig. 3b). Co-incubation with FFAs and 5 ug/ml UT or okadaic acid enhanced glucose uptake (Fig. 3c) compared to vehicle only treatment.

### Ceramide levels and PP2A protein activity and expression in skeletal muscle

**Figure 4:**
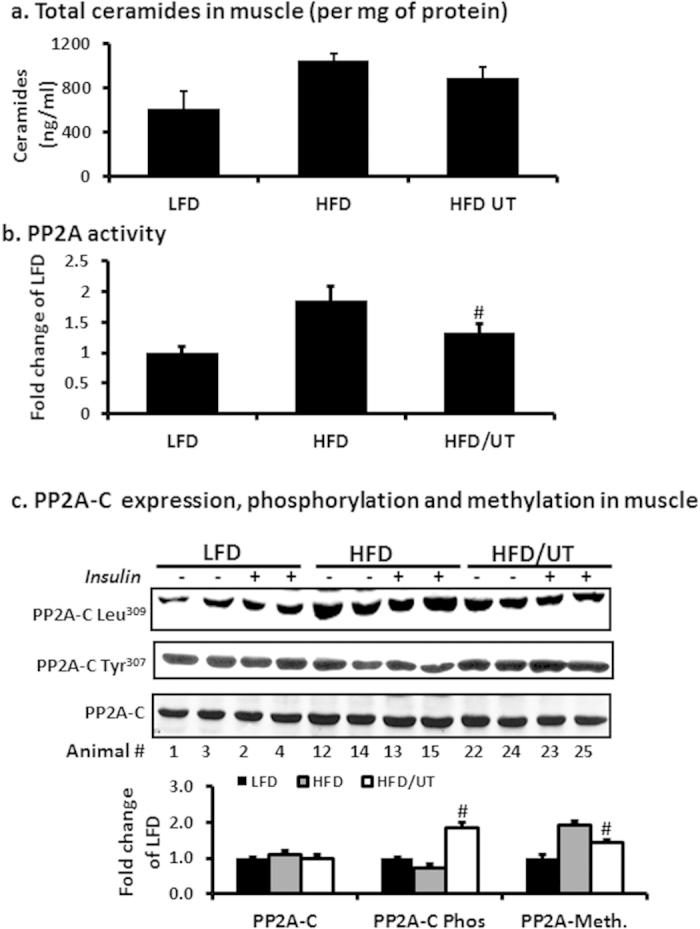
UT extract attenuates HFD induced hyperactivity of PP2A and enhances Akt phosphorylation in skeletal muscle. Male C57BL/6J mice were fed were fed the experimental diets for 12 weeks. After sacrifice **(a)** 30 mg of gastrocnemius muscle was extracted for lipids by the Folch partition and ceramide quantities determined by tandemn mass spectrometry (LC/MS/MS). **(b)** 30 mg muscle was homogenized and lysates used to determine phosphatase PP2A activity by a standard kit (EMD Millipore, Temecula CA). **(c)** Protein homogenates from 20 mg gastrocnemius muscle were separated by SDS-PAGE and analyzed by immune blot analysis. Representative blots for PP2A- C protein expression, PP2A-C tyrosine phosphorylation and PP2A-C leucine methylation are shown. Fold change relative to LFD alone for each protein was calculated and mean ± SEM graphed (n = 11). ^#^denotes significant difference with HFD (P < 0.05).

As expected, skeletal muscle of the HFD group accumulated ceramides and had significantly increased level of PP2A activity compared to the LFD group. UT supplementation did not lower ceramides significantly (Fig. 4a) but lowered PP2A activity significantly compared to the HFD (P < 0.01; Fig. 4b). Protein expression of PP2A subunits A and B between the three groups was not different (data not shown). Expression of PP2A subunit C was also not different but phosphorylation level of PP2A-C on Tyr^307^, an indicator of PP2A inactivation, was lowered in the HFD group when compared to LFD. UT supplementation enhanced this phosphorylation significantly compared to the HFD group (P < 0.01; Fig. 4c). Methylation of PP2A-C on Leu^309^, also an indicator of PP2A catalytic activity, was higher in the HFD group compared to LFD. UT supplementation in the HFD significantly lowered this methylation (P < 0.01; Fig. 4c).

### Ceramide levels and PP2A protein activity and expression in cultured myotube

**Figure 5:**
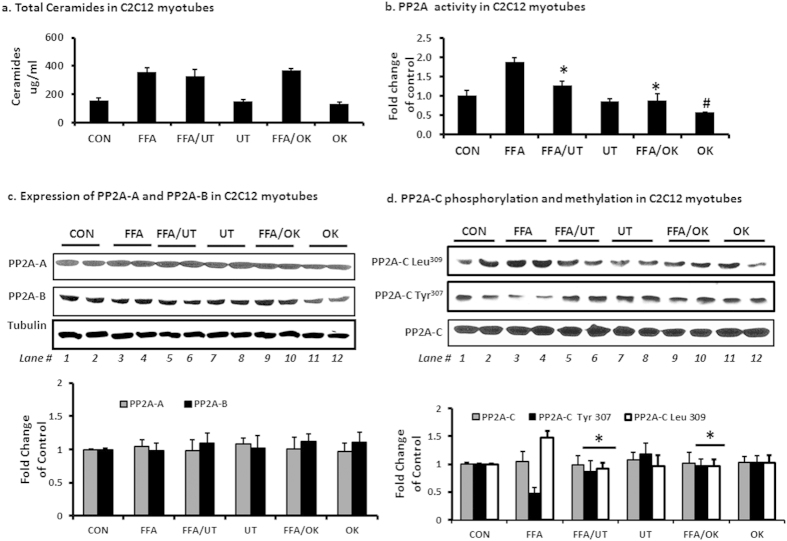
UT extract attenuates FFA induced hyperactivity of PP2A and enhances Akt phosphorylation in C2C12 myotubes. Fully differentiated C2C12 myotubes were incubated with palmitate (250 uM) with or without UT (5 ug/ml) or 10 nM okadaic acid (OK) for 16 hours. **(a)** Cells were extracted for lipids by the Folch partition and ceramide quantities determined by tandemn mass spectrometry (LC/MS/MS). **(b)** Cell lysates were used to determine PP2A activity by a standard kit (EMD Millipore, Temecula CA, USA). **(c)** Cell lysates were separated by SDS-PAGE and analyzed by immune blot analysis for PP2A-A and PP2A-B protein expression and **(d)** PP2A-C protein expression, PP2AC tyrosine phosphorylation and PP2A-C leucine methylation. Each panel represents an experiment independently repeated three times on different batches of myotubes. ^*^denotes significant difference with FFA only treatment (P < 0.05). ^#^denotes significant difference with the untreated control treatment (P < 0.05).

As expected, exposure of myotubes to FFAs increased ceramide levels by more than one fold (Fig. 5a) and enhanced PP2A activity (Fig. 5b). Co-incubation with FFAs and UT or okadaic acid had no effect on ceramide accumulation but lowered PP2A activity compared to the FFA treatment only (P < 0.01; Fig. 5a,b). FFAs and UT had no effect on protein expression of PP2A subunits A, B and C (Fig. 5c,d). However, FFAs significantly lowered PP2A-C phosphorylation on Tyr^307^ and enhanced methylation on Leu^309^ (P < 0.01; Fig. 5d lanes 1 and 2 vs lanes 3 and 4). Co-incubation of FFAs with UT enhanced PP2A-C Tyr^307^phosphorylation and lowered Leu^309^ methylation compared to FFA treatment alone (P < 0.01; Fig. 5d; lanes 3 and 4 vs lanes 5 and 6). Incubation with UT only enhanced PP2A-C Tyr^307^phosphorylation compared to the vehicle only treatment (P < 0.01; Fig. 5d; lanes 1 and 2 vs. lanes 7 and 8).

## Discussion

In this study, we report the effects of a UT extract on plasma glucose homeostasis and insulin signaling in skeletal muscle tissues from mice fed a high-fat diet. Consistent with prior reports in rats^10^,^11^,^12^,^13^, we show that supplementing a high fat diet with UT enhances glucose homeostasis. Several studies have demonstrated that excess dietary lipids results in accumulation of lipid metabolites particularly ceramides that impair insulin signaling in insulin sensitive tissues^5^,^6^,^7^,^19^,^20^,^21^. To date, there are no reported studies on the effects of UT on skeletal muscle insulin sensitivity. We show that UT supplementation enhances skeletal muscle insulin sensitivity despite the accumulation of ceramides. Furthermore, UT attenuated FFA induced insulin resistance in myotubes by enhancing Akt phosphorylation despite the accumulation of ceramides. We further show that the enhanced Akt phosphorylation is accompanied with a reduction in HFD or FFA induced hyperactivity of PP2A, the phosphatase that is activated by ceramides and is responsible for dephosphorylating Akt.

The connection between insulin resistance and obesity is well established. Obesity leads to elevated levels of circulating FFAs that contribute to insulin resistance by promoting excessive deposition lipid metabolites in tissues not suited for fat storage such as skeletal muscle^5^,^6^,^7^,^18^,^19^,^20^. Here in we report on the effects of UT on glucose homeostasis and insulin signaling in skeletal muscle of a robust pre-diabetes model; high fat fed C57BL/6J mice. These mice developed mild to moderate hyperglycemia, post- glucose load hyperglycemia, fasting hyperinsulinemia, and a diminished insulin response after 12 weeks feeding. Supplementation of the HFD with UT improved glucose homeostasis and insulin sensitivity as assessed by, IPIT and HOMA-IR without affecting food intake, body weight or body composition (Tables 1 and 2; Fig. 1).

We evaluated the effects of UT in skeletal muscle because it is the site of majority of insulin-stimulated glucose disposal and insulin resistance in skeletal muscle can be evident decades before β-cell failure and overt hyperglycemia^22^. Supplementation of the HFD with UT abated insulin resistance as evidenced by enhanced levels of Akt phosphorylation on Ser^473^ and Thr^308^ (Fig. 2). Akt is a serine/threonine kinase that mediates many effects of insulin including glucose uptake and metabolism. Akt activation depends on regulatory mechanisms that require dual phosphorylation on Ser^473^ and Thr^308^ by PDK1 and the TORC2 complex respectively and translocation to the plasma membrane^15^,^16^,^20^,^21^. By moderating these effects on Akt, UT enhanced insulin sensitivity in skeletal muscle. The *in vitro* data complemented the *in vivo* data by showing that UT attenuated the inhibitory effects of FFAs on Akt phosphorylation at Ser^473^ and Thr^308^ and insulin stimulated glycogen synthesis; an essential downstream function that results from the phosphorylation and inactivation of GSK-3 by Akt. Activated GSK-3 reduces glycogen synthesis and plays a dominant role in inducing skeletal muscle insulin resistance^24^. UT enhanced glucose uptake in both FFA treated and control cells. We included a treatment with okadaic acid (OK) a naturally occurring polyether that enhances Akt phosphorylation by inhibiting PP2A. UT effects were comparable to those of OK in attenuating the ability of FFAs to induce insulin resistance in skeletal muscle cells (Fig. 3).

Among lipid metabolites that accumulate with high fat diets, ceramides have been shown to be the most active in negatively regulating insulin signaling by blocking the activation of Akt without affecting upstream signaling events^5^,^6^,^7^,^19^,^20^,^21^. Akt is dependent on stimulatory signals from the insulin receptor for activation but is simultaneously under negative control by phosphatases particularly PP2A^15^,^16^,^17^,^21^,^25^. Studies in insulin responsive cells, have implicated hyperactivity of PP2A in the pathogenesis of Akt dephosphorylation, leading to attenuation of glucose transport stimulation^17^,^20^. PP2A is a serine/threonine phosphatase that leaves Akt dephosphorylated and unable to translocate to the plasma membrane. In contrast to other lipids, ceramides have been shown to be specific activators of PP2A^25^. Besides activating PP2A, ceramides that accumulate in caveolin-enriched domains of the cell membrane also interfere with PKCzeta catalyzed phosphorylation of Akt thus preventing it from participating in downstream insulin receptor signaling^7^. Insulin inhibits PP2A activity but this effect is diminished in diabetic models^25^. Our data shows that UT attenuated the ability of accumulated ceramides to induce PP2A hyperactivity without lowering ceramide levels in muscle tissue and C2C12 myotubes (Figs 4 and 5). We concluded that inhibition of PP2A activity in skeletal muscle by UT plays a role in the significant increase in Akt phosphorylation observed despite the increased levels of ceramides.

The major finding and one that requires a mechanism is UT how reduces PP2A activity. We therefore evaluated UT effects on PP2A expression and posttranslational modification. PP2A is a heterotrimeric complex comprised of a structural or scaffolding subunit A, a regulatory subunit B, and a catalytic subunit C^17^,^26^,^27^. It controls the phosphorylation of a wide range of substrates but gains specificity through the targeting regulatory subunit B^21^,^25^. Phosphorylation of the catalytic subunit at Tyr^307^ results in substantial reduction of activity^17^,^21^,^25^. The catalytic subunit is also subject to methyl esterification of its C terminus on Leu-^309^. This methylation activates the enzyme. Inhibiting methylation contributes to a decrease in activity^25^,^27^. We show that the enhanced PP2A activity in HFD mice muscle resulted from increased Leu^309^ methylation and decreased Tyr^307^ phosphorylation of the catalytic subunit. UT supplementation reduced Leu^309^ methylation and increased Tyr^307^ phosphorylation thus decreasing overall activity of the enzyme (Fig. 4). In C2C12 myotubes, UT attenuated FFA induced PP2A methylation resulting in lower levels of enzyme activity without affecting protein expression (Fig. 5). Okadaic acid, a known PP2A inhibitor, decreased FFA induced PP2A hyperactivity by more than 50% while UT decreased it by about 32% resulting in an increased relative amount of phosphorylated Akt. However, it is difficult to compare activity because the bioactive components of UT extract are unknown. Our data suggests that a contributing mechanism, by which UT enhances insulin sensitivity in skeletal muscle in the presence of excess dietary lipids, is modulation of PP2A activity.

A limitation of our study is that we have not elucidated the bioactive components of UT. Identification of the active components of UT will be an important next step for determining its potential as a metabolically beneficial botanical supplement for obesity induced insulin resistance. Since stinging nettle has a history of human use as a food, the effects of its components are unlikely to pose toxicity issues. This was corroborated in this study. Based on work done using the whole extract we conclude that UT components attenuate obesity induced insulin resistance through mechanisms that enhance Akt phosphorylation and its downstream effects without affecting food intake, body weight or body composition.

## Additional Information

**How to cite this article**: Obanda, D. N. *et al.* An extract of *Urtica dioica* L. mitigates obesity induced insulin resistance in mice skeletal muscle via protein phosphatase 2A (PP2A). *Sci. Rep.* **6**, 22222; doi: 10.1038/srep22222 (2016).

## Figures and Tables

**Table 1:** Food intake, body weight, body composition and circulating FFAs.

	LFD	HFD	HFD/UT
Food intake per day (grams)	2.56 ± 0.81	2.23 ± 0.49	2.26 ± 0.61
Body weight (grams)			
Week 0	22.02 ± 0.45	21.82 ± 0.46	22.08 ± 0.18
Week 6	25.68 ± 0.44	30.40 ± 0.70	29.63 ± 0.88
Week 12	27.99 ± 0.60	35.68 ± 1.15	34.98 ± 1.11
Body composition (% Fat)			
Week 0	14.96 ± 0.50	15.56 ± 1.06	15.43 ± 0.89
Week 6	17.33 ± 0.99	25.03 ± 1.48	27.08 ± 2.18
Week 12	16.23 ± 1.14	28.38 ± 1.14	29.58 ± 2.02
Body composition (% Muscle)			
Week 0	70.63 ± 0.15	69.91 ± 0.09	70.35 ± 0.23
Week 6	69.64 ± 0.25	63.07 ± 0.21	62.05 ± 0.13
Week 12	69.58 ± 0.21	60.90 ± 0.31	59.54 ± 0.20
Circulating FFAs (week 12) (mmol/l)	0.39 ± 0.1	0.45 ± 0.03	0.44 ± 0.09

**Table 2:** Values of Area under the curve.

	LFD	HFD	HFD/UT
Glucose tolerance test (IPGT)	69,508 ± 1101	97,860 ± 2103	85,808 ± 1903
Insulin tolerance test (IPIT)	23,878 ± 967	34,258 ± 1105	27,440 ± 589*

^*^significantly different with HFD.

## References

[b1] Reaven G. M. Banting lecture 1988. Role of insulin resistance in human disease. Diabetes. 37, 1595–1607 (1988). 3056758 10.2337/diab.37.12.1595

[b2] Kahn B. B. & Flier J. S. Obesity and insulin resistance. J Clin Invest. 106 (**4**), 473–481 (2000). 10953022 10.1172/JCI10842 PMC380258

[b3] McLellan K. C. P., Wyne K., Villagomez E. T. & Hsueh W. A. Therapeutic interventions to reduce the risk of progression from prediabetes to type 2 diabetes mellitus. Ther Clin Risk Manag. 10, 173–188 (2014). 24672242 10.2147/TCRM.S39564 PMC3964168

[b4] Rood R. C. *et al.* Effects of Artemisia species on *de novo* lipogenesis in vivo. Nutrition. 30 (**7–8**), S17S20 (2014). 10.1016/j.nut.2014.03.029 PMC4120075 24985100

[b5] Obanda D. N. *et al.* Bioactives of *Artemisia dracunculus* L. mitigate the role of ceramides in attenuating insulin signaling in rat skeletal muscle cells. Diabetes. 61 (**3**), 597–605 (2012). 22315320 10.2337/db11-0396 PMC3282822

[b6] Kraegen E. W., Cooney G. J., Ye J. M., Thompson A. L. & Furler S. M. The role of lipids in the pathogenesis of muscle insulin resistance and beta cell failure in type II diabetes and obesity. Exp Clin Endocrinol Diabetes. 109, S189–20 (2001). 11460570 10.1055/s-2001-18581

[b7] Blouin C. M. *et al.* Plasma membrane subdomain compartmentalization contributes to distinct mechanisms of ceramide action on insulin signaling. Diabetes. 59, 600–610 (2010). 19959757 10.2337/db09-0897 PMC2828662

[b8] Riddle M. C. Glycemic management of type 2 diabetes: an emerging strategy with oral agents, insulins and combinations. Endocrinol Metab Clin North Am. 34, 77–98 (2005). 15752923 10.1016/j.ecl.2004.12.002

[b9] Stafylas P. C., Sarafidis P. A. & Lasaridis A. N. The controversial effects of thiazolidinediones on cardiovascular morbidity and mortality. Int J Cardiol. 131 (**3**), 298–304 (2009). 18684530 10.1016/j.ijcard.2008.06.005

[b10] Farzami B., Ahmadvand D., Vardasbi S., Majin F. J. & Khaghani S. Induction of insulin secretion by a component of *Urtica dioica* leave extract in perifused Islets of Langerhans and it’s *in vivo* effects in normal and streptozotocin diabetic rats J. Ethnopharmacol. 89 (**1**), 47–53 (2003). 14522431 10.1016/s0378-8741(03)00220-4

[b11] Bnouham M. *et al.* Antihyperglycemic activity of the aqueous extract of *Urtica dioica*. Fitoterapia. 74 (**7–8**), 677–681 (2003). 14630172 10.1016/s0367-326x(03)00182-5

[b12] Golalipour M. J. & Khori V. The protective activity of *Urtica dioica* leaves on blood glucose concentration and beta-cells in streptozotocin-diabetic rats. Pak J. Biol Sci. 10 (**8**), 1200–1204 (2007). 19069917 10.3923/pjbs.2007.1200.1204

[b13] Domola M. S. *et al.* Insulin mimetics in *Urtica dioica*: Structural and computational analyses of *Urtica dioica* extracts. Phytother. Res. 24, S175–S182 (2010). 20013820 10.1002/ptr.3062

[b14] Chavez J. A. *et al.* A role for ceramide but not diacylglycerol, in the antagonism of insulin signal transduction by saturated fatty acids. J. Biol Chem. 278, 10297–10303 (2003). 12525490 10.1074/jbc.M212307200

[b15] Kuo Y. C. *et al.* Regulation of phosphorylation of Thr-308 of Akt, cell proliferation, and survival by the B55alpha regulatory subunit targeting of the protein phosphatase 2A holoenzyme to Akt. J. Biol Chem. 283, 1882–1892 (2008). 18042541 10.1074/jbc.M709585200

[b16] Ugi S. *et al.* Protein phosphatase 2A negatively regulates insulin’s metabolic signaling pathway by inhibiting Akt (protein kinase B) activity in 3T3-L1 adipocytes. Mol Cell Biol. 24 (**19**), 8778–89 (2004). 15367694 10.1128/MCB.24.19.8778-8789.2004 PMC516764

[b17] Janssens V. & Goris J. Protein phosphatase 2A: a highly regulated family of serine/threonine phosphatases implicated in cell growth and signaling. Biochem. J. 353, 417–439 (2001). 11171037 10.1042/0264-6021:3530417 PMC1221586

[b18] Wang Z. Q. *et al.* Effects of dietary fibers on weight gain, carbohydrate metabolism, and gastric ghrelin gene expression in mice fed a high-fat diet. Metabolism. 56, 1635–42 (2007). 17998014 10.1016/j.metabol.2007.07.004 PMC2730183

[b19] Stratford S., Hoehn K. L., Liu F. & Summers S. A. Regulation of insulin action by ceramide: Dual mechanisms linking ceramide accumulation to the inhibition of Akt/protein kinase B. J. Biol Chem. 279, 36608–36615 (2004). 15220355 10.1074/jbc.M406499200

[b20] Wan X. & Helman L. J. Levels of PTEN protein modulate Akt phosphorylation on serine 473, but not on threonine 308, in IGF-II-overexpressing rhabdomyosarcomas cells. Oncogene. 22, 8205–8211 (2003). 14603261 10.1038/sj.onc.1206878

[b21] Galbo T. *et al.* PP2A inhibition results in hepatic insulin resistance despite Akt 2 activation. Aging. 5 (**10**), 770–81 (2013). 24150286 10.18632/aging.100611 PMC3838779

[b22] Defronzo R. A. & Tripathy D. Skeletal muscle insulin resistance is the primary defect in type 2 Diabetes. Diabetes Care. 32, suppl 2, S157–S163 (2009). 19875544 10.2337/dc09-S302 PMC2811436

[b23] Draznin B. Molecular mechanisms of insulin resistance: serine phosphorylation of insulin receptor substrate-1 and increased expression of p85alpha: the two sides of a coin. Diabetes. 55 (**8**), 2392–7 (2006). 16873706 10.2337/db06-0391

[b24] Boden G. & Shulman G. I. Free fatty acids in obesity and type 2 diabetes; defining their role in the development of insulin resistance and β-cell dysfunction. Euro J. Clin Invest. 32 (Suppl. 3), 14–23 (2002). 10.1046/j.1365-2362.32.s3.3.x 12028371

[b25] Dobrowsky R. T., Kamibayashi C., Mumby M. C. & Hannun Y. A. Ceramide activates heterotrimeric protein phosphatase 2A. J. Biol Chem. 268 (**21**), 15523–15530 (1993). 8393446

[b26] Lee J., Chen Y., Tolstykh T. & Stock J. A specific protein carboxyl methylesterase that demethylates phosphoprotein phosphatase 2A in bovine brain. Proc Natl Acad Sci USA 93, 6043–6047 (1996). 8650216 10.1073/pnas.93.12.6043 PMC39185

[b27] Guénin S. *et al.* PP2A activity is controlled by methylation and regulates oncoprotein expression in melanoma cells: A mechanism which participates in growth inhibition induced by chloroethylnitrosourea treatment. Int. J. Oncology. 32 (**1**), 49–57 (2008). 18097542

